# Serological response to Epstein-Barr virus early antigen is associated with gastric cancer and human immunodeficiency virus infection in Zambian adults: a case-control study

**DOI:** 10.11604/pamj.2016.23.45.8503

**Published:** 2016-02-18

**Authors:** Violet Kayamba, Mwaka Monze, Akwi Wasi Asombang, Kanekwa Zyambo, Paul Kelly

**Affiliations:** 1 Tropical Gastroenterology & Nutrition Group, University of Zambia School of Medicine, Nationalist Road, Lusaka, Zambia; 2 University Teaching Hospital, Nationalist Road, Lusaka, Zambia; 3 Division of Gastroenterology and Hepatology, University of Missouri-Columbia School of Medicine, MO, USA; 4 Blizard Institute, Barts & The London School of Medicine and Dentistry, Turner Street, London, UK

**Keywords:** Gastric cancer, Epstein-Barr virus, HIV, EBNA-1, EBV- EA

## Abstract

**Introduction:**

Gastric cancer is one of the major causes of cancer related deaths, but data from sub-Saharan Africa are very scanty. The cancer genome atlas (TCGA) initiative confirmed Epstein-Barr virus (EBV) related cancer as a distinct subtype, and we set out to look for serological evidence of its role in a sub-Saharan African patient group.

**Methods:**

We used stored serum samples obtained from a gastric cancer case-control study conducted between 2010 and 2012 in Lusaka, Zambia. A total of 147 patients were included with 51 gastric adenocarcinoma cases and 96 age and sex matched controls. The presence of antibodies to EBV nuclear antigen-1 (EBNA-1) and early antigen (EA) was determined using commercially available ELISA kits. Data were analysed in STATA Stata Corp, College Station TX.

**Results:**

Over 90% of all the samples analysed were positive for antibodies to EBNA-1. The presence of antibodies to EBV EA was significantly higher in gastric cancer cases than in controls, (OR 4.38; 95% CI 1.53-13.06, P = 0.0027), with an attributable risk of 23%. HIV infection was also associated with EBV EA seroprevalence (OR 10.97; 95% CI 2.26 -13.06, P = 0.001) but not EBNA-1 (OR 0.81; 95% CI 0.10 -38.75, P = 0.596). There was no association of EBV infection with age below 45 years, *Helicobacter pylori* infection, intestinal metaplasia, gastric atrophy or inflammation.

**Conclusion:**

We therefore conclude that EBV exposure is common among Zambian adults and that EBV EA seropositivity is associated with gastric cancer and HIV infection, but not premalignant lesions.

## Introduction

Gastric cancer is one of the major causes of cancer related deaths globally [1]. The burden of cancer related health problems is increasing in Africa, although it continues to receive relatively low public health priority [2]. Gastric cancer data from Sub-Saharan Africa are scarce [3] and there is an urgent need to begin collecting these data in order to understand the risk factors. There is an increase in the incidence of gastric cancer in sub-Saharan countries such as Uganda [4] in contrast with the reported decline in developed countries [5]. Estimates of the epidemiology of gastric cancer in Zambia also show an increase in the number of cases especially among young adults. It however, remains unclear if this is a real increase or just a reflection of improving awareness and availability of better diagnostic facilities.

Epstein-Barr virus (EBV) is a DNA oncogenic herpes virus present in about 90% of the global adult population [6]. It is associated with nasopharyngeal and gastric cancers, follicular dendritic cell tumors/sarcomas, Burkitt's lymphoma, lymphomatoid granulomatosis, pyothorax-associated lymphoma and Human Immunodeficiency Virus (HIV) associated conditions such as hairy leukoplakia and central nervous system lymphomas [7]. The Cancer Genome Atlas (TCGA) project recently characterized gastric adenocarcinoma into four major subtypes: EBV-positive tumors, microsatellite unstable tumors, genomically stable tumors, and tumors with chromosomal instability [10]. There is evidence that up to 9% of gastric cancer is attributed to EBV infection [8, 9]. The analysis however, did not include samples from indigenous African subjects nor was the influence of Human Immunodeficiency virus (HIV) infection on the molecular characterization of gastric cancer considered. As a result the distribution of TCGA subtypes in sub-Saharan Africa is not very clear.

Gastric cancer in Zambia presents at younger ages than in Europe or North America [11]. It is not known how much EBV infection influences the development of gastric cancer in these young adults. Recent data from the USA suggest there has been an increase in the occurrence of non-cardia gastric cancer among young adults [12]. The interaction between HIV infection and EBV in gastric cancer development has not been described, although it has been reported that HIV infection increases the risk for EBV acquisition in Zambian children [13] and there is evidence of a higher EBV replication in HIV infected compared to uninfected children [14]. Higher loads of EBV DNA have also been reported in HIV infected individuals. In addition, HIV infected patients are at an increased risk of developing cancers with a known infectious cause [15] and it is uncertain if this is true for EBV positive gastric cancer. In this study, we evaluated the association between gastric cancer and EBV infection in Zambian adults using stored samples collected from a previous case-control study [16]. We evaluated the presence of antibodies to EBV nuclear antigen-1 (EBNA-1) and early antigens (EA), using enzyme-linked immunosorbent assay (ELISA). Antibodies against EBNA-1 are usually not detected until 90 days after an acute infection [6] and may persist for life [17]. EA antibodies are produced during the early phase of lytic replication and may be present in up to 80% of actively infected individuals [6].

## Methods

This was a retrospective analysis of stored serum samples. The samples were collected during a case-control study on gastric adenocarcinoma conducted at the University Teaching Hospital (UTH) in Lusaka, between November 2010 and January 2012 [16]. UTH is the largest referral hospital in Zambia attending to patients from all parts of the country. In this study, patients referred to the unit for diagnostic upper gastrointestinal endoscopy were considered for enrolment. Those willing to participate signed consent forms and were enrolled either as cases or controls. Gastric biopsies were obtained from all lesions suspected of being malignant. Cases were defined as patients with histologically confirmed gastric adenocarcinoma, while controls were age and sex matched patients with dyspeptic symptoms but no evidence of gastric cancer. Biopsies for histopathology were taken from the controls as follows, two each from the cardia, body and antrum. Patients with gastric Kaposi's sarcoma or lymphoma were excluded. 51 cases and 96 controls were studied. Blood was taken from study participants and serum was extracted and stored at negative 80 ° C within our laboratories at UTH. The samples were clearly labelled with the date of collection and identification (ID) number. Ethical approval was obtained from the University of Zambia Biomedical and Ethics Committee (reference number 009-08-13).

### Laboratory analysis

All the stored serum samples were retrieved although two samples (1 case and 1 control) were insufficient for analysis. Serum was then allowed to thaw to room temperature. Following the manufacturer's instructions, we used Platelia, EB-NA-1 IgG for qualitative determination of IgG antibodies to EBNA-1 (Trinity Biotech, Wicklow, Ireland), and Platelia, EBV-EA-D IgG (Trinity Biotech, Wicklow, Ireland) for qualitative determination of IgG antibodies to EA. The Immune Status Ratio (ISR) was calculated by dividing the optical density for each specimen by the cut-off calibrator value. The assays were all validated as advised by the manufacturer. VK and KZ carried out the ELISAs and one full plate was repeated to confirm the consistency of the results. ISR values of less than 0.90 were considered negative, those between 0.91 and 1.09 considered equivocal and those above 1.10 were declared positive. Equivocal results were not included in the final analysis.

### Statistical analysis

We used STATA 13 (Stata Corp, College Station TX) to analyse the data. For continuous variables showing a non- Gaussian distribution, the Kruskal-Wallis test was used to compare the two groups. For categorical variables, the Fisher's exact test was used. Odds Ratios with 95% confidence intervals, and two sided P values were derived to define the frequency of risk factors in cases and controls. Probability values less than 0.05 were considered statistically significant. Stepwise logistic regression was used to assess the relative contributions of different exposure variables to the risk of gastric cancer.

## Results

A total of 147 serum samples (51 cases and 96 controls) were retrieved. Three cases had equivocal results, one for EBNA-1 and three for EA. One case was equivocal both for EBNA-1 and EA. Among the controls, two were equivocal for EA and one for EBNA-1. Serum samples with equivocal results were excluded from the final results. Therefore, when analysing for EBV antibodies, 48 cases and 93 controls were used. The baseline characteristics of the two samples were similar, with the exception of body mass index, which was significantly lower in gastric cancer patients (Table 1).

**Table 1:** Basic demographic characteristics of cases and controls showing that the two groups were comparable

	Cases, n = 51 n (%)	Controls, n = 96 n(%)	P
**Females**	21(41.2)	48(51.6)	0.296
**Age in years:**			0.238
Less than 45	10(19.6)	27(29.0)	
Greater or equal to 45	41(80.4)	66(71.0)	
**Educational achievement:**			0.207
None	10(19.6)	10(10.8)	
Primary or higher	41(80.4)	83(89.2)	
**Occupation:**			0.833
None	10(19.6)	21(22.6)	
Formal or informal	41(80.4)	72(77.4)	
**Body Mass Index (kg/m**^**2**^**)**			0.001
Less than 20	22(43.1)	16(17.2)	
More or equal to 20	29(56.9)	77(82.8)	

### EBNA -1 and EA antibody expression in cases and controls

92.9% of all the patients (both cases and controls) were positive for antibodies to EBNA-1, and there was minimal difference between cases and controls (Table 2). We compared seropositivity to EBNA-1 and EA between cases and controls and found that the proportion of gastric cancer patients with positive EA was significantly higher than in controls (Table 2). The attributable risk of gastric cancer in patients with positive EA antibodies was 22.5%. The group with evidence of past infection but no reactivation was the least likely to have gastric cancer, and the group with evidence of current infection was the most likely to have gastric cancer (Table 3).

**Table 2:** The comparison of antibody expression between cases and controls. Its shows that there were significantly more patients with EBV-EA-D IgG among the gastric cancer cases

	Cases n = 48(%)	Controls n = 93(%)	OR; 95% CI	P
EBV EB-NA-1 IgG positive	43 (89.6)	88 (94.6)	0.49; 0.11 -2.26	0.3084
EBV-EA-D IgGpositive	14 (29.2)	8 (8.6)	4.38; 1.53 -13.06	0.0027

**Table 3:** Summary of the serological classification of cases and controls, with probable interpretations

	Cases n (%)	Controls n (%)	OR; 95% CI	P	Possible interpretation
EBNA-negative and EA-negative	3 (6.2)	5 (5.4)	0.17(1.17-6.34)	1.000	No infection
EBNA-negative and EA-positive	2 (4.2)	0 (0)	-	0.1143	Early primary infection
EBNA-positive and EA-negative	31 (64.6)	79 (86.0)	0.30(0.12-0.74)	**0.0046**	Past infection without reactivation
EBNA-positive and EA-positive	12 (25.0)	8 (8.6)	3.54(1.20-10.8)	**0.0112**	Probable viral reactivation

### Risk factors for EBV in both patient groups

We then analysed the association of antibodies to EBV with probable risk factors (Table 4). None of the factors considered showed any association with antibodies to EBNA-1 (Table 4). 90% of the HIV infected patients and 92% of the HIV negative patients were positive for EBNA-1, with no significant difference between the two. In contrast, 60% of those with HIV infection were positive for EA compared to 12% of the HIV uninfected patients (Odds ratio 10.97; 95% CI 2.26 to 57.26, P = 0.001; Table 4). EBV antibodies were not associated with gastric inflammation (acute or chronic), low pepsinogen 1 to 2 ratio or intestinal metaplasia.

**Table 4:** Probable risk factors for EBV infection in this group of patients, with HIV positive patients having significantly more individuals with EBV-EA-D IgG

	EBV EB-NA-1 IgG n = 141		EBV-EA-D IgG n = 141	
	OR; 95% CI	P	OR; 95% CI	P
Age below 45 years	1.05; 0.24 - 6.38	1.000	1.08; 0.32- 3.23	1.000
HIV positive	0.81; 0.10 -38.75	0.596	10.97; 2.26 - 57.26	0.001
*H.pylori* positive	0.57; 0.01 -4.37	1.000	1.67; 0.35 -15.95	0.738
Low pep 1:2 ratio	2.10; 0.49 -12.6	0.362	1.30; 0.46 -3.62	0.637
Low gastrin	0.34; 0.08 -1.76	0.111	0.18; 0.00 -1.27	0.126
Intestinal metaplasia	0.33; 0.04 -4.19	0.238	0.74; 0.01 -6.61	1.000
Acute or active inflammation	0.43; 0.05 -3.48	0.374	0.28; 0.00 -2.40	0.427
Chronic inactive inflammation	0.62; 0.05 -4.63	0.692	0.42; 0.06 -2.36	0.284

### EBV anti-early antigen seropositivity by anatomical site and histological type

We analysed the anatomical sites of the tumour as determined at endoscopy: 13% were in the cardia, 23% in the body, 52% in the antrum and 12% were in more than one site. There was no association between the site of the tumour and the presence of EBV EA antibodies (data not shown, P = 0.66). We also considered the type of gastric cancer, using the Lauren classification. Among the patients included in this analysis, 81% had intestinal type, 13% had diffuse type, and 4% had mixed type of gastric adenocarcinoma. The classification for one tumour (2%) was not available. There was no association between the type of tumour and the presence of EBV EA antibodies (data not shown, P = 0.52).

## Discussion

The development of gastric cancer in young Zambian adults is not well described. We set out to investigate the association between gastric cancer and EBV infection by measuring antibodies to EBNA-1 and EA in gastric cancer patients with age and sex matched controls. We found an association with antibodies to EA but not to EBNA-1. We also found that HIV infection itself was a risk factor for EA seropositivity. Gastric cancer is one of the major gastrointestinal cancers diagnosed in Zambia and the prognosis tends to be very poor with significant mortality at one year. [18] We recently completed a retrospective audit of endoscopy records going back almost four decades. The findings showed that the occurrence of gastric cancer is increasing in young adults, but not in older patients. This is in line with reports from the USA which also showed an increase in non cardia gastric cancer incidence among whites below the age of 40 years, emphasising that it is important to define independent trends in cancer subtypes which may be responding to differential exposures [12]. The diagnosis of gastric cancer in poor resource countries such as Zambia remains a challenge due to inadequate diagnostic facilities. It is likely that the burden of gastric cancer in this region remains under estimated. EBV is an almost ubiquitous infection globally resulting in clinically significant disease in a few individuals. EBV associated gastric tumours tend to confer better patients outcomes than those not associated with EBV [19]. It is not clear how much influence EBV infection has on the development of gastric cancer in Zambia. The TCGA consortium analysis confirms the importance of EBV in gastric cancer, but despite the well-known high prevalence of EBV in sub-Saharan Africa, very little work has been done. To our knowledge, this was the first study looking at the association of EBV infection with gastric cancer in Zambia. We used ELISA to determine the presence of antibodies to EBNA-1 and EA. EBNA-1 develops much later during the course of an infection and can persist for life. However, EBNA-1 antibodies may be undetectable in the sera of up to 5% healthy individuals with past exposure to EBV [20].

In our study, 90% of the cases’ and 95% of the controls’ sera was positive for anti-EBNA-1 without any significant difference between the groups. There have been reports of anti-EBNA-1 loss during immunosuppression, [20] but we found no loss of anti-EBNA-1 in HIV infection. EA signifies EBV recent infection or reactivation. It is a transient antibody and can disappear with 3 to 4 months of infection or reactivation. It is not very clear how many transient phases of reactivation EBV infected persons have during their lifetime, as there are no distinctive clinical symptoms. We found that antibodies to EA were significantly higher in gastric cancer patients, with an attributable risk of 21.7%. This suggests that EBV continues replicating after the development of gastric cancer, findings that have also been suggested by other investigators [8, 21]. It is yet to be established whether EBV reactivation is the one that triggers gastric carcinogenesis or the changes associated with gastric carcinogenesis trigger the reactivation of previously latent virus. Interpretation of antibodies to EA should be cautious, as they are only present in about 80% of affected individuals. There is currently no consensus on the threshold titres of EA for signifying EBV re-activation. There have been reports of an interrelationship between *Helicobacter pylori (H. pylori)* infection and EBV. Lima et al reported finding H. pylori infection in all EBV positive gastric tumours. [22] Our study did not demonstrate any such association, as there was no significant difference in EBV seropositivity between patients with and without antibodies to H. pylori infection. The incidence of EBV associated gastric cancer tends to decrease with advancing age. We found no difference in EBV antibodies between older and younger patients. Some investigators have reported an association between EBV infection and gastric premalignant lesions [23]. While others have failed to demonstrate this [24] Schetter et al suggested that EBV reactivation occurs as an early phase of gastric carcinogenesis [23]. We found no evidence of an association between gastric intestinal metaplasia and either EBNA-1 or EA antibodies. There is a need to further explore the role of EBV infection in gastric malignancy as identification of the point at which it plays a role might suggest opportunities for prevention. We found no evidence that EBV EA antibodies vary by tumour site: cardia, body or antrum. The TCGA project identified a preponderance of EBV associated tumours in the body [10]. van Beek J and others found that the EBV associate tumours were more in the proximal regions of the stomach [25]. While our sample size is limited, our findings do not support an association between EBV with any particular site. We now have evidence that most of gastric adenocarcinoma in Zambia is of the intestinal type. Again, we found no evidence of an association between the Lauren classification and EBV infection. We recognize the fact that using serology to assess the association between gastric cancer and EBV infection is not optimal, but we have identified an important association and our data suggest that a high proportion of Zambian cancers could be EBV-related (population attributable fraction 23%). Further information from in-situ hybridization or PCR would give additional information as to the activation or latency of any virus present. This study has demonstrated the urgent need for more gastric cancer research in sub-Saharan Africa, a region heavily affected by HIV infection. The role of HIV infection and its interaction with other viruses on the development of either premalignant lesions or gastric cancer may be different here to the role played in other geographical settings.

## Conclusion

Serological response to Epstein-Barr virus early antigen is associated with gastric cancer and HIV infection. This association does not apply to gastric premalignant lesions.

### What is known about this topic

- Gastric cancer is one of the leading causes of cancer related mortality and 9% of all the cases can be attributed to Epstein-Barr virus (EBV) infection. There is an increase in gastric cancer diagnosis among Zambians less than 60 years, and the age at first diagnosis is lower than expected. The occurrence of gastric cancer in these young adults cannot be entirely attributed to a young population structure.

### What this study adds

- This study has showed that the occurrence of gastric cancer in Zambia is associated with antibodies to EBV early antigens but not EBV nuclear antigen-1. The results also suggest an influence of EBV re-activation on gastric cancer carcinogenesis in Zambia. An association between EBV early antigens and HIV infection has been demonstrated.
